# Lateral entorhinal cortex lesions impair both egocentric and allocentric object–place associations

**DOI:** 10.1177/2398212820939463

**Published:** 2020-07-14

**Authors:** Maneesh V. Kuruvilla, David I. G. Wilson, James A. Ainge

**Affiliations:** 1 School of Psychology and Neuroscience, University of St Andrews, St Andrews, UK; 2 Wicking Dementia Research and Education Centre, University of Tasmania, Hobart, TAS, Australia

**Keywords:** Hippocampus, spatial memory, associative, episodic memory, navigation, medial entorhinal cortex, cognitive map, landmarks

## Abstract

During navigation, landmark processing is critical either for generating an allocentric-based cognitive map or in facilitating egocentric-based strategies. Increasing evidence from manipulation and single-unit recording studies has highlighted the role of the entorhinal cortex in processing landmarks. In particular, the lateral (LEC) and medial (MEC) sub-regions of the entorhinal cortex have been shown to attend to proximal and distal landmarks, respectively. Recent studies have identified a further dissociation in cue processing between the LEC and MEC based on spatial frames of reference. Neurons in the LEC preferentially encode egocentric cues while those in the MEC encode allocentric cues. In this study, we assessed the impact of disrupting the LEC on landmark-based spatial memory in both egocentric and allocentric reference frames. Animals that received excitotoxic lesions of the LEC were significantly impaired, relative to controls, on both egocentric and allocentric versions of an object–place association task. Notably, LEC lesioned animals performed at chance on the egocentric version but above chance on the allocentric version. There was no significant difference in performance between the two groups on an object recognition and spatial T-maze task. Taken together, these results indicate that the LEC plays a role in feature integration more broadly and in specifically processing spatial information within an egocentric reference frame.

## Introduction

Spatial memory and navigation require us to learn and remember the locations of landmarks within our environment. These landmarks can take numerous forms from large geographical features to small objects within our local environment. We can use landmarks to form an allocentric map of the external world that allows flexible navigation, including the generation of shortcuts; the cognitive map (O’Keefe and Nadel, 1978; Tolman, 1948). We can also use them to support egocentric representations of the world that are used during processes such as path integration (McNaughton et al., 2006). In recent years, our understanding of how navigation and spatial memory mechanisms are represented in the brain has evolved rapidly, and the circuits supporting both egocentric and allocentric representations are becoming more well understood.

Place cells in the hippocampus fire in consistent locations relative to landmarks providing a potential neural mechanism to support the cognitive map (O’Keefe and Dostrovsky, 1971). Place cells receive input from two major input pathways from the medial (MEC) and lateral (LEC) entorhinal cortices (Van Strien et al., 2009). Recent studies of MEC have demonstrated a number of clearly spatially modulated signals. These include grid cells (Hafting et al., 2005), head direction cells (Sargolini et al., 2006), border cells (Barry et al., 2006; Solstad et al., 2008), conjunctive cells (Sargolini et al., 2006), and object vector cells (Høydal et al., 2019). These spatial signals are all tied to landmarks, although landmarks in these studies are represented by a range of stimuli from distal room cues to objects close in proximity to the animal.

Studies of LEC have shown a clear lack of spatially modulated signals (Hargreaves et al., 2005; Yoganarasimha et al., 2011), although there is the suggestion that there is weak spatial tuning in LEC to local cues within the environment (Neunuebel et al., 2013). This is supported by studies showing that some LEC neurons are tuned to objects (Deshmukh et al., 2012; Deshmukh and Knierim, 2011; Tsao et al., 2013). Based on these findings, it has been suggested that distal global cues could be processed by MEC, while proximal local cues within the immediate environment are processed by LEC and that these two reference frames are tied together in the hippocampus to enable spatial memory and navigation (Knierim et al., 2014; Wang et al., 2020). Consistent with this suggestion, disruption of MEC results in deficits in spatial learning and memory when tests use global cues (Steffenach et al., 2005; Tennant et al., 2018; Van Cauter et al., 2013) while disruption of LEC impairs learning of a spatial memory task based on local cues (Kuruvilla and Ainge, 2017).

However, all of the studies covered so far involve testing the ability to use landmarks to support allocentric spatial memory. Recent studies have demonstrated that LEC neurons show clear egocentric coding while MEC signals are dominated by allocentric cues when rats are foraging in an open environment with no local cues (Wang et al., 2018). In this study, we asked whether there is a critical role for the LEC in using landmarks to support spatial memory based on either egocentric or allocentric frames of reference. To test the suggestion that LEC is specifically involved in egocentric encoding of space, we examined the effect of LEC lesions on rats’ ability to remember the associations between objects and locations in situations where egocentric and allocentric reference frames were encouraged. For comparison, we examined how these animals performed on a reward-based non-associative spatial task on the T-maze to test the hypothesis that LEC may have a general role in spatial processing. Given that this task can be solved using either an egocentric or allocentric strategy, deficits would suggest an inability to use either type of spatial framework.

## Methods

### Subjects

Male Lister Hooded rats (Envigo, Bicester, UK) were housed in groups of four on a 12-h light/dark cycle (*n* = 14; average weight at start of experiment: 359 g). Behavioural testing was conducted 5 days a week during the light cycle. The maintenance of laboratory animals and their use in scientific experiments complied with national (Animals (Scientific Procedures) Act, 1986) and international (European Communities Council Directive of 24 November 1986 (86/609/EEC)) legislation governing the maintenance of laboratory animals and their use in scientific experiments. Local approval was also received from the St Andrews Animal Welfare and Ethics Committee. For both experiments, animals had free access to water while in their cages. Specifically for experiment 2, animals were food restricted to no less than 85% of their free feeding bodyweight. This was done to motivate animals during behavioural testing, which involved a food reward.

## Apparatus: experiment 1

### Object recognition and object–place associative recognition tasks

**Figure 1.**
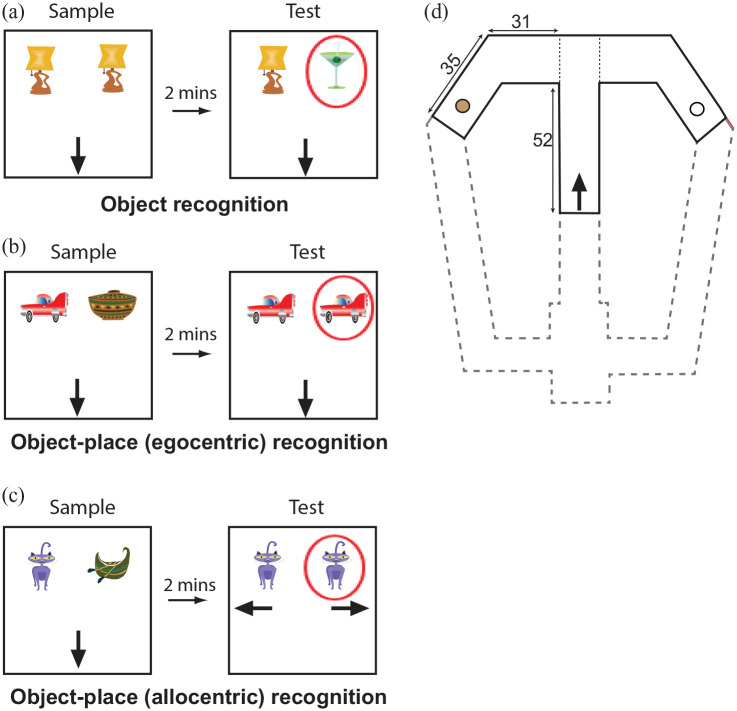
Behavioural task designs. ((a)–(c)) Schematic depicting the structure of a given trial within each behavioural task used in experiment 1. Different novel objects were used each day. Red circles indicate the object/association that is novel. Black arrows indicate the position and direction that the rat was placed in the box at the start of each trial. (d) Schematic showing the design of the modified T-maze used in experiment 2. The area of the maze in grey large dashed lines was blocked off from the rat. The black arrow indicates the position and direction that the rat was placed on the maze at the start of each trial. Circles represent small wells for food rewards. All measurements shown are in cm.

Three recognition memory tasks were conducted: standard object recognition (﻿OR) and two versions of object–place (OP) recognition promoting an egocentric and allocentric strategy respectively. All three tasks were run using two objects placed in a 67 cm square box with 40 cm high walls. The box could be set up as two different ‘contexts’ by swapping in/out the walls and floor of the box. The ‘white’ context had floor and wall inserts painted white. In the ‘stripes’ context, the walls and floor inserts were painted with black and white vertical stripes (5 cm width) with an additional plastic-coated metal mesh overlaid on the floor. The two objects were attached to the box floor with Dual Lock Velcro (3M, St Paul, MN), side-by-side approximately 15 cm apart and towards the north wall (see Figure 1(a)). Objects used were three-dimensional (3D) household items made from ceramic, metal, glass or plastic that were easy to clean between trials and were approximately the size of the rat (in at least one dimension). The box itself was situated on a platform 32 cm above the ground and encircled by a black curtain. Prominent extra-maze cues were attached to the curtain.

## Apparatus: experiment 2

### Spatial T-maze task

For the spatial task, a modified T-maze with 3 cm high walls was set up 80 cm from the floor. The maze had a central stem that extended 60 cm to the T-junction before turning off to the left and right arms. Only a portion of the central stem was used for the experiment (see Figure 1(a)). The maze was open to the testing room, providing animals with prominent extra-maze cues. The experimenter stood in the same place towards the base of the central stem at the start of every trial to serve as a salient extra-maze cue.

### Behavioural procedure: experiment 1

Following 1 week of extensive handling to habituate the rats to the experimenter, rats were individually habituated to contexts (4 days) and then to novel objects within contexts (4 days). The two context configurations were not relevant to the current hypotheses, and no significant effects of context were found on task performance (data not reported). The use of the two contexts and order of presentation of contexts was counterbalanced within and across tasks for both groups of rats. Rats were then tested on a series of recognition tasks in the following order: OR, OP (egocentric) and OP (allocentric). Each task was run for 4 days, with rats receiving a sample and test trial on each of those days. At the end of the sample trial, rats were placed in a holding cage for 1 min while the box was cleaned and configured for the test trial.

### OR task

In the sample trial, rats were given 3 min to explore two identical objects (e.g. toy lamps). The trial ended at the end of 3 min or when rats explored both objects for 15 s each, whichever was shorter. During the test trial (3-min duration), rats were exposed to a new copy of the previously seen object (e.g. toy lamp) as well as a novel object not previously seen (e.g. martini glass). Memory for the objects from the sample trial would be expected to drive preferential exploration of the novel object (e.g. martini glass) in the test trial. Identity and position of the novel object were counterbalanced within and across days for the lesion and sham groups. The experimenter placed the rat in the box facing the south wall at the start of both trials (see Figure 1(a)).

### OP tasks

In the sample trial, rats were exposed to two different objects (e.g. toy car, clay pot) for 3 min. During the test trial (3-min duration), two identical copies of one of the previously seen objects (e.g. toy cars) were placed in the box for rats to explore. Integrated memory of object and location from the sample trial would be expected to drive preferential exploration of the object in the location in which it had not previously been experienced in the test trial. Two versions of the OP task were run, in which either an egocentric or allocentric encoding of space was encouraged. The key difference between the egocentric and allocentric tasks was the direction in which rats were placed at the start of the test trial (see Figure 1(b) and (c)). For the egocentric task, and similar to the OR task, rats were always placed facing the south wall. However, for the allocentric task, rats began the test trial facing either the east or west walls. This version of the task has previously been used to promote an allocentric encoding of space (Langston and Wood, 2010). Initial heading direction and choice and location of novel object were counterbalanced within and across days for the lesion and sham groups.

## Behavioural procedure: experiment 2

### Spatial T-maze task

Rats were habituated to the T-maze for 3 days. On the first day, rats were placed at the base of the central stem and allowed to explore the maze for 5 min. On the second day, a food reward (one-half of a cereal loop, Weeto™) was placed in both the east and west arms of the maze. Rats were placed in the starting point and allowed to explore the maze until they had found and eaten both rewards or 10 min had elapsed, whichever happened sooner. On the third day, a food reward was only placed in the west arm. Rats were allowed to find and eat the food reward in the west arm and also explore the non-rewarded east arm.

After completing habituation, rats were trained on a spatial task for 7 days, receiving four trials per day. Rats were trained to turn left on to the west arm to receive a food reward (see Figure 1(d)). In every trial, rats were placed at the base of the central stem and were free to choose to explore east or west arms. If the rat chose the west arm, the trial was ended after the rat had consumed the food reward. If the rat chose the east arm, then the rat was immediately removed from the maze. In between trials, rats were placed in a holding cage while the maze was cleaned and the reward replaced.

### Surgery

Group sizes were determined based on previous studies showing robust effect sizes for rats performing OR and OP tasks in our laboratory (see also statistics and results sections for details of analysis of generalisability of findings to larger samples). Rats in both the lesion (*n* = 6) and sham (*n* = 8) groups were initially anaesthetized using isoflurane (Abbot Laboratories Ltd., Maidenhead, UK) in an induction box. They were then placed in a stereotaxic frame (David Kopf, Tujunga, CA, USA) where anaesthesia was maintained via a facemask mounted on the incisor bar (2%–3% isoflurane, 1.2 l/min O_2_). A pre-surgical analgesic Rimadyl (0.05 ml/rat; 5% w/v carprofen; Pfizer Ltd, Kent, UK) was administered subcutaneously. After shaving the animal’s scalp, a midline incision was made and holes drilled bilaterally at stereotaxic co-ordinates targeting LEC: −6.5 mm from Bregma; ±4.5 mm from the midline (measured on the skull surface). Dura was cut using the bent tip of a 30-gauge needle and the pipette lowered into the brain at a 10° angle to 6.4 mm below dura. For animals in the lesion group, 188 nl of ibotenate (0.03M solution in sterile phosphate buffer; Sigma-Aldrich, UK) was infused by pressure ejection from a drawn glass micropipette (tip diameter 30–40 microns) and left in situ for 5 min after infusion. Sham controls underwent the identical procedure receiving only the vehicle solution (sterile phosphate buffer). Rats were given 7 days to recover from surgery before behavioural testing began.

### Perfusion

Rats were humanely euthanised with intraperitoneal injections of 200 mg/ml/kg sodium pentobarbitone (‘Dolethal’, Univet, Bicester, UK) and transcardially perfused with phosphate-buffered saline (0.9%). This was followed by at least 250 ml of paraformaldehyde solution (4% made up in 0.1% phosphate buffer solution). Brains were then extracted and placed overnight in 20% sucrose solution (made up in 0.1% phosphate buffer).

### Histology

Brains were immersed in egg yolk within 24-well tissue culture plates containing paraformaldehyde (40%) in the empty neighbouring wells. These were left for 5 days to allow the egg to fix onto the outside of the brains. Brains were subsequently cut into 50 µm coronal sections on a freezing microtome and then mounted 1:4 sections onto slides. Sections were then stained on the slides with cresyl violet. To do this, slides were placed in a slide holder and then submerged in glass vases of xylene (2 min), 100% alcohol (1 min), 50% alcohol (1 min), water (1 min), cresyl violet (2 min), running water (5 min), 50% alcohol (1 min), 100% alcohol (1 min) and finally cleared in xylene. Slides were individually removed from xylene and coverslipped using DPX mountant (BDH Laboratory Supplies, Poole, UK).

### Lesion analysis

Slides were viewed under a light microscope (Leitz Diaplan) at magnification ×10 and ×4. The extent of lesioned area was judged by the lack of cell bodies or by cells that were shrunken and damaged. Lesion damage was drawn onto 10 standardised sections of LEC with reference to Paxinos and Watson (2007; ranging from −7.66 to −4.42 mm) using Scion Image (v4.0.3.2).

## Behavioural analyses

### Discrimination ratio

For the three recognition memory tasks, animals were scored to be actively exploring an object when their noses were within 2 cm of the object. The exploration times for the two objects were then converted into discrimination ratios (discrimination ratio = (time at novel object – time at familiar object) / (time at novel object + time at familiar object)) to determine an animal’s relative exploration of the novel versus familiar object or OP association. The discrimination ratio calculated here is equivalent to the D2 measure used by Dix and Aggleton (1999). For each task, discrimination ratios were calculated for each day and then an average across the 4 days used for analysis. To check for reliability, a separate observer, who was blind to condition, re-scored a subset of videos for each task, and these scores were found to be consistently within 10% of the experimenter’s.

### Accuracy and latency measures

For each trial on the spatial T-maze task, animals were judged to have made a choice when all four of their paws were simultaneously beyond the entrance to either the left or right arms. Animals taking a left turn were judged to have made a correct choice while those turning right were classified as making an incorrect choice. Response latencies were also measured on each trial by recording how long (in seconds) it took animals to make a correct or incorrect choice from the time they were placed at the base of the central stem.

### Statistical analysis

In experiment 1, separate univariate analyses of variance (ANOVAs) were conducted on the discrimination ratios and exploration rates in the test phase and sample phase for each of the three recognition memory tasks. To determine the likelihood of the reported effects persisting across larger samples, we ran data analysis with bootstrap-coupled estimation (Ho et al., 2019). A total of 5000 bootstrap samples were taken; the confidence interval is bias-corrected and accelerated. For each permutation *P* value, 5000 reshuffles of the control and LEC groups were performed. The *P* value reported is the likelihood of observing the effect size, if the null hypothesis of zero difference is true. Figures 3 and 4 along with the statistical analyses presented in Table 1 were generated from an open-source website (www.estimationstats.com; Ho et al., 2019). One-sample *t* tests were also used to assess whether the average discrimination ratios for the lesion and sham groups were different to chance (0) on the various recognition memory tasks. Additional paired-samples *t* tests were conducted for both groups to compare discrimination ratios between the first and second halves of the OP allocentric task. In experiment 2, 2 × 7 mixed ANOVAs, with lesion group (LEC; sham) as the independent factor and training day (days 1–7) as the repeated measures factor, were performed for both accuracy and latency during the spatial T-maze task. One-sample *t* tests were conducted to assess whether accuracy performance for the lesion and sham groups was different to chance (0.5) for each test day. Bonferroni corrections were applied to post hoc comparisons conducted on significant main and interaction effects. A Greenhouse–Geisser correction was applied in instances where the sphericity assumption was violated for the repeated measures factor (training day). Statistical analyses were performed using IBM SPSS Statistics 25.0^®^.

**Table 1.** Summary *P* values and observed effect sizes for differences in discrimination ratios between the two groups across the three recognition memory tasks. Columns 2 and 3 indicate *P* values and observed effect sizes based on the original data set. Columns 4 and 5 indicate *P* values and observed effect sizes for 5000 reshuffles of the sham and LEC groups across the three recognition memory tasks.

Task	Univariate ANOVA	Partial η^2^	Two-sided permutation *t* test	Unpaired Cohen’s *d* between sham (*n* = 8) and lesion (*n* = 5) groups
Object recognition	*P* = .061	$\eta_{p}^{2}$= .204	*P* = .132	95.0% CI: −0.96 (−2.79, −0.15)
OP recognition (egocentric)	*P* = .023	$\eta_{p}^{2}$ = .316	*P* = .049	95.0% CI: −1.29 (−2.92, 0.01)
OP recognition (allocentric)	*P* = .003	$\eta_{p}^{2}$ = .526	*P* = .003	95.0% CI: −1.99 (–3.18, −0.98)

OP: object–place; ANOVA: analysis of variance; CI: confidence interval.

## Results

### Histology

**Figure 2.**
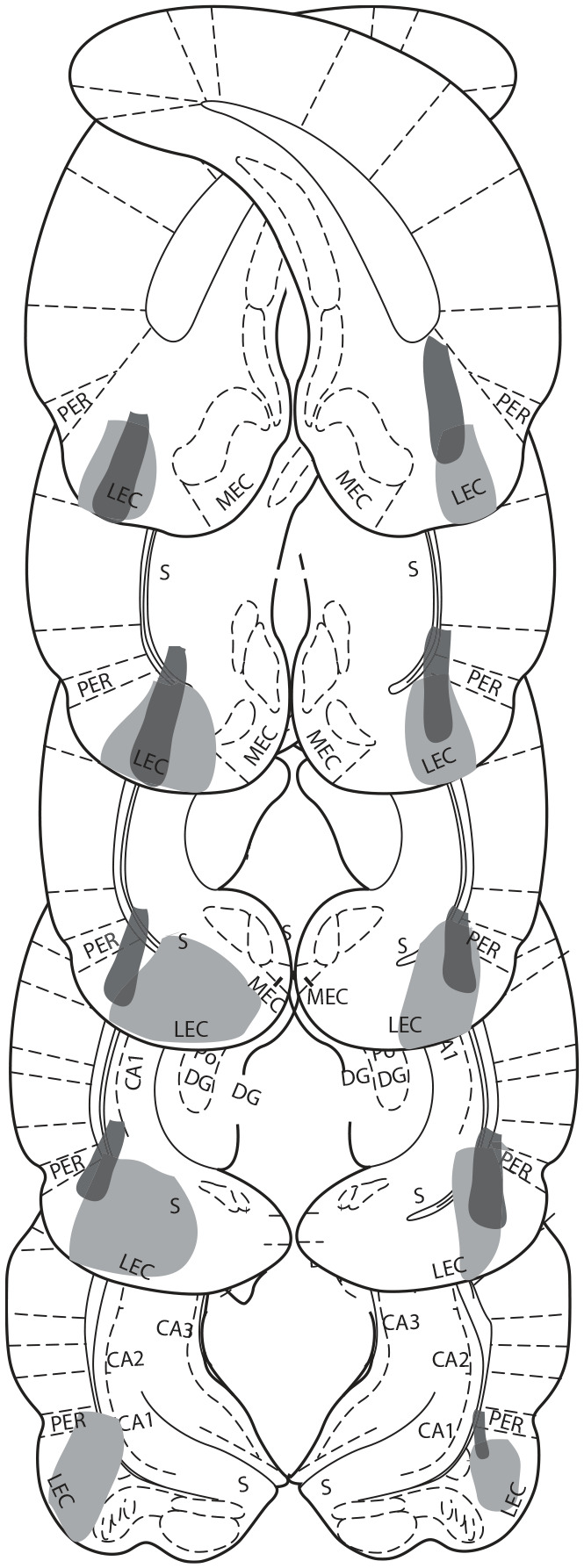
Schematic representation of lesion damage extent. Representations of coronal sections adapted from Paxinos and Watson (2007) are at −7.64, −7.04, −6.72, −6.3, −5.8 mm from Bregma, from top to bottom, respectively. Grey colouring represents lesion damage to LEC where the rat with the greatest lesion damage is shown in light grey and the rat with the least damage in dark grey.

Histological analysis determined that five of six rats in the lesion group had successful bilateral lesions of the LEC. Lesion damage from rats with the largest and smallest lesions is depicted in Figure 2. One rat had a unilateral lesion of LEC. We have previously shown that unilateral LEC damage can produce similar deficits to bilateral LEC lesions (Wilson et al., 2013a, 2013b) and so analyses were carried out with and without this animal. Analyses presented exclude this animal except where its inclusion did produce changes to significance levels and these differences are highlighted. For transparency, the unilateral lesioned rats’ data point is specifically labelled in figures. In most rats, there was some minor damage to ventral subiculum, CA1 and/or perirhinal cortex but this was estimated at being less than 5% of the overall volume of those structures. Rats in sham group had no lesion damage.

## Behavioural analysis: experiment 1

### LEC lesions do not impair simple object recognition

**Figure 3.**
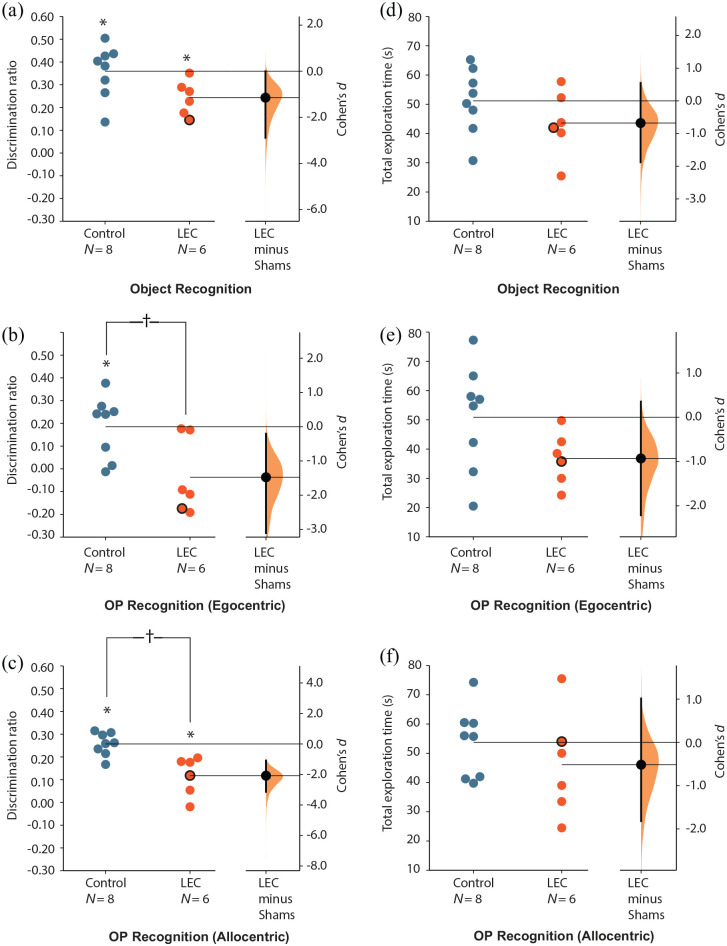
Recognition memory of LEC lesion and sham rats in experiment 1. The Cohen’s *d* difference between LEC and sham is shown in the above Gardner-Altman estimation plot. Both groups are plotted on the left axes; the group mean difference is plotted on floating axes on the right as a bootstrap sampling distribution. The mean difference is depicted as a dot; the 95% confidence interval is indicated by the ends of the vertical error bar. ((a)–(c)) Discrimination ratios across the three recognition memory tasks. ((d)–(f)) Total exploration time across the three recognition memory tasks. One rat with unilateral lesion damage is represented with a black outline. Asterisks represent discrimination ratios significantly different to chance following a one-sample *t* test (*t* test vs 0; *p* < .05). Crosses represent significantly different average discrimination ratios between lesion and sham groups (*p* < .05).

Analysis of performance on the object recognition (OR) task replicated our previously reported effects (Kuruvilla and Ainge, 2017; Vandrey et al., 2020; Wilson et al., 2013a, 2013b). Average discrimination ratios were not significantly different between sham and LEC lesion rats (*F*_(1,11)_ = 2.82, *p* = .061, $\eta_{p}^{2}$ = .204; Figure 3(a)). In addition, rats in both the sham (*t*_(7)_ = 8.76, *p* < .001) and lesion (*t*_(4)_ = 8.93, *p* < .001) groups had discrimination ratios significantly greater than chance. Thus, rats in both sham and lesion groups were able to remember objects. There was no significant difference in the total amount of time exploring objects in general (time spent at novel + familiar objects) between rats in sham and LEC lesion groups (*F*_(1,11)_ = 1.19, *p* = .149, $\eta_{p}^{2}$ = .098); Figure 3(d)).

### LEC lesions impair egocentric-based OP associative recognition

As both sham and lesioned rats were able to demonstrate memory for familiar objects, we could now assess their performance on tasks that required them to remember the association of objects and the locations in which they were experienced. Analysis of performance on the OP (egocentric) recognition task also revealed effects that replicated those we have previously reported (Wilson et al., 2013b). Average discrimination ratios were significantly different between groups with LEC lesioned animals performing significantly worse than shams (*F*_(1,11)_ = 5.09, *p* = .023, $\eta_{p}^{2}$ = .316; Figure 3(b)). Rats in the sham group had discrimination ratios significantly greater than chance (*t*_(7)_ = 3.82, *p* = .004), demonstrating that they preferred exploring novel OP associations and therefore had remembered familiar OP associations. In contrast, rats in the LEC lesion group showed no such preference for exploring novel OP associations (*t*_(4)_ = –0.81, *p* = .470), showing chance-level performance. There was no significant difference in the total amount of time exploring objects between rats in sham and LEC lesion groups in either the sample (*F*_(1,11)_ = 1.24, *p* = .289, $\eta_{p}^{2}$ = .101) or test phases (*F*_(1,11)_ = 2.37, *p* = .076, $\eta_{p}^{2}$ = .177); Figure 3(e)). Overall, these findings are consistent with previous evidence indicating the role of the LEC in OP associative recognition memory.

### LEC lesions impair allocentric-based OP associative recognition

Similar to the egocentric version of this task, average discrimination ratios were significantly worse for LEC lesioned animals relative to shams (*F*_(1,11)_ = 12.23, *p* = .003, $\eta_{p}^{2}$ = .526; Figure 3(c)). However, unlike in the egocentric version, rats with LEC lesions were able to recognise OP associations above levels of chance (*t*_(4)_ = 2.76, *p* = .025), as were rats with sham lesions (*t*_(7)_ = 14.43, *p* < .001). Thus, both groups recognised familiar OP associations in the test phase. There was no significant difference in the total amount of time exploring objects between rats in sham and LEC lesion groups in either the sample (*F*_(1,11)_ = 0.03, *p* = .870, $\eta_{p}^{2}$ = .003) or test phases (*F*_(1,11)_ = 1.13, *p* = .156, $\eta_{p}^{2}$ = .093); Figure 3(f)).

**Figure 4.**
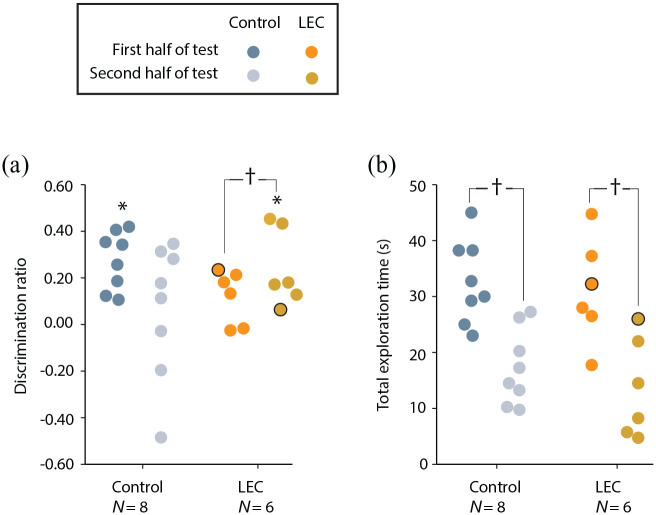
Performance of LEC lesion and sham rats on OP allocentric task split across two halves of testing sessions. (a) Discrimination ratios. (b) Total exploration time. One rat with unilateral lesion damage is represented with a black outline. Asterisks represent discrimination ratios significantly different to chance following a one-sample *t* test (*t* test vs 0; *p* < .05). Crosses represent significantly different average discrimination ratios between lesion and sham groups (*p* < .05).

To examine the deficit seen in the LEC lesion group in more detail, we compared discrimination ratios between the first and second halves of the test sessions (Figure 4(a)). One possibility is that rats with LEC lesions initially try and use an egocentric strategy, which would be ineffective, and only slowly adapt to using an allocentric strategy as the test trial continues. Paired-samples *t* tests revealed that LEC lesioned rats had significantly higher discrimination ratios in the second half of sessions compared with the first (*t*_(4)_ = –3.47, *p* = .013) suggesting that these animals were only able to recognise the position of objects within an allocentric framework after some time in the environment. This was supported by one-sample *t* tests, which revealed that the LEC lesion group was performing at chance in the first half of sessions (*t*_(4)_ = 1.95, *p* = .062) but above chance during the second half of sessions (*t*_(4)_ = 3.90, *p* = .009). Sham-lesioned animals did not have significantly different discrimination ratios between the two testing halves (*t*_(7)_ = 1.71, *p* = .066). Interestingly, one-sample *t* tests revealed that sham animals performed above chance in the first half of sessions (*t*_(7)_ = 6.23, *p* < .001) but at chance in the latter half (*t*_(7)_ = 0.64, *p* = .271). As would be expected, this suggests that normal animals quickly recognise the position of objects within an allocentric framework and explore the novel configuration in the early stages of the trial with discrimination becoming less prominent through the course of the trial as the novel OP configuration becomes more familiar.

These results should also be considered in the context of total exploration times for the two groups. Animals in both the sham (*t*_(7)_ = 3.87, *p* = .003) and LEC lesion groups (*t*_(4)_ = 10.86, *p* < .001) spent considerably more time exploring objects in first half relative to the second half of test sessions (Figure 4(b)), which would be anticipated as the objects become more familiar. So it is not the case that the general motivational drive to explore objects has been altered by LEC lesions. Rather, it suggests animals’ exploratory drive decreases in the normal way in both groups, but as the LEC lesioned animals spend more time in the environment, their ability to encode objects in an allocentric framework improves and so despite spending less time exploring the objects, their discrimination of novel and familiar OP configurations improves.

Overall, rats with LEC lesions remained impaired relative to shams on this OP associative recognition memory task. However, promoting the use of an allocentric spatial framework improved the ability of rats with LEC lesions to recognise an object within a previously experienced location. Furthermore, this ability to recognise an object within a previously experienced location emerges towards the end of the test trial, suggesting that it takes rats with lesions of the LEC longer to place themselves within an allocentric spatial framework.

### Reproducibility of findings

We went on examine the likelihood of the reported effects persisting across larger samples. This was done using bootstrap-coupled estimation (Ho et al., 2019). A total of 5000 bootstrap shuffled samples were used to create permutation *P* values, where *P* is the likelihood of observing the effect size, if the null hypothesis of zero difference is true. Table 1 illustrates that the findings are robust with the shuffled data confirming the significant group differences in both OP tasks but not in the OR tasks.

## Behavioural analysis: experiment 2

### LEC lesions do not impair non-associative spatial memory

Given that rats were impaired at both types of OP recognition task, we went on to examine whether LEC lesions produced a deficit in a standard test of spatial reference memory on a T-maze. Critically, this task does not require rats to integrate items with spatial locations and rather is a non-associative test of spatial memory. Our previous studies have demonstrated that LEC is necessary for the association of features of an event suggesting that non-associative spatial memory should not be affected. LEC animals were not impaired in learning this simple spatial task. Interestingly, the rats with LEC lesions were more accurate than the sham animals at the beginning of training. This was confirmed with a 2 × 7 mixed ANOVA that revealed significant main effects of group (*F*_(1,11)_ = 23.23, *p* = .001, $\eta_{p}^{2}$ = .679) and day (*F*_(6,66)_ = 3.02, *p* = .011, $\eta_{p}^{2}$ = .216) as well as a significant group × day (*F*_(6,66)_ = 2.39, *p* = .037, $\eta_{p}^{2}$ = 179) interaction. Post hoc univariate ANOVAs revealed that LEC lesioned animals were significantly more accurate than shams on days 1 (*F*_(1,11)_ = 22.85, *p* = .001, $\eta_{p}^{2}$ = .675) and 2 (*F*_(1,11)_ = 5.03, *p* = .047, $\eta_{p}^{2}$ = .314). Post hoc repeated measures ANOVAs revealed a significant difference in accuracy performance across days for the sham (*F*_(6,42)_ = 6.92, *p* < .001, $\eta_{p}^{2}$ = .497) but not the LEC lesion group (*F*_(2.79,11.17)_ = 0.57, *p* = .638, $\eta_{p}^{2}$ = .124). One-sample *t* tests further highlighted a difference in accuracy between the two groups on the first 2 days of training. LEC lesioned animals showed above-chance accuracy (day 1: *t*_(4)_ = 6.53, *p* = .003; day 2: *t*_(4)_ = 3.50, *p* = .025) while animals in the sham group remained at chance level performance (day 1: *t*_(7)_ = 1.53, *p* = .170; day 2: *t*_(7)_ = 1.43, *p* = .197). Both groups showed above-chance accuracy on the last 5 days of training.

**Figure 5.**
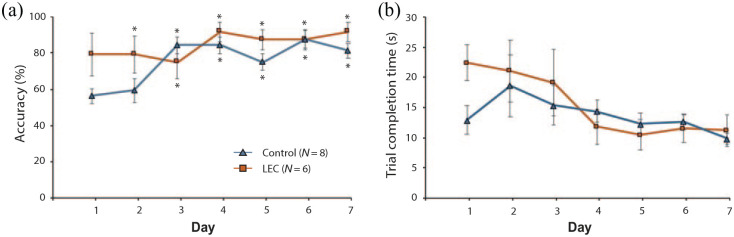
Performance of LEC lesion and sham rats during a non-associative spatial task on a modified T-maze. (a) Average correct responses across 7 training days. (b) Average trial completion time from the rat being placed on the maze to touching reward across 7 training days (four trials per day). Asterisks represent accuracy performance significantly above chance following a one-sample *t* test (*t* test vs 0.5; *p* < .05).

With the inclusion of the unilateral LEC lesion animal, a 2 × 7 mixed ANOVA revealed a significant main effect of day (*F*_(6,72)_ = 4.10, *p* = .001, $\eta_{p}^{2}$ = .255) but neither a main effect of group (*F*_(1,12)_ = 4.57, *p* = .054, $\eta_{p}^{2}$ = .276) nor a day × group (*F*_(6,72)_ = 1.78, *p* = .116, $\eta_{p}^{2}$ = .129) interaction effect. Post hoc pairwise comparisons on the main effect of ‘Day’ did not reveal a significant difference in accuracy between training days for either the sham or lesion groups. One-sample *t* tests highlighted a difference in accuracy between the two groups during the initial days of training (see Figure 5(a)). Both groups showed at-chance accuracy on training day 1 (LEC: *t*_(5)_ = 2.45, *p* = .058, shams: *t*_(7)_ = 1.53, *p* = .170). However, on day 2, the LEC lesion group was performing above chance (*t*_(5)_ = 2.91, *p* = .034) while the shams remained at chance levels of accuracy (*t*_(7)_ = 1.43, *p* = .197). Shams reached above-chance performance by day 3 (*t*_(7)_ = 7.51, *p* < .001), matching the lesion group (*t*_(5)_ = 2.74, *p* = .041). Both groups showed above-chance accuracy on training days 3 to 7.

Overall, both sets of analyses indicate that LEC lesioned animals were unimpaired on a non-associative spatial memory task and actually showed improved accuracy relative to shams towards the beginning of training.

### LEC lesions do not impair spatial response latencies

Response latencies were compared between the two groups across training days as a proxy for evaluating decision-making certainty and motivation towards the food reward. Figure 5(b) shows that both groups decreased their response latency across testing. A 2 × 7 mixed ANOVA revealed a significant main effect of day (*F*_(2.81,30.94)_ = 3.23, *p* = .039, $\eta_{p}^{2}$ = .227) but neither a main effect of group (*F*_(1,11)_ = 0.16, *p* = .701, $\eta_{p}^{2}$ = .014) nor a day × group (*F*_(2.81,30.94)_ = 1.24, *p* = .311, $\eta_{p}^{2}$ = .101) interaction (Figure 5(b)). Post hoc pairwise comparisons revealed that both groups were faster at accurately completing trials on day 7 compared to day 1 (*M* = –8.77, *SE* = 1.98, *p* = .021). These results indicate that both the LEC lesion animals and shams were similarly motivated and decisive in completing the spatial task. In addition, the change in latency between the first and last training day for both groups suggests that animals were learning the task over time, as expected. The significance of the data did not change when the animal with the unilateral lesion was included.

## Discussion

The hippocampal–entorhinal network has been shown to be critical for spatial memory (Ainge et al., 2006, 2007; Ainge and Langston, 2012; Andersen et al., 2006; Broadbent et al., 2004; Martin and Clark, 2007; Morris et al., 1982; Save and Sargolini, 2017; Steffenach et al., 2005; Van Cauter et al., 2013). Recent studies, however, have suggested that different parts of the network are tuned to either egocentric or allocentric frames of reference with LEC showing clear egocentric spatial tuning (Wang et al., 2018). We have previously shown that LEC is critically important for integrating features of an event, including the locations of objects within an environment (Kuruvilla and Ainge, 2017; Wilson et al., 2013a, 2013b). Here, we tested the role of LEC in integrating objects within either an egocentric or allocentric frame of reference. This was assessed using two versions of the OP recognition task: one in which animals were introduced to the environment from a consistent spatial location to encourage an egocentric frame of reference, the other in which animals are introduced from multiple different locations encouraging an allocentric frame of reference. Rats would have to first situate themselves in more global, allocentric space before being able to make an associative memory judgement as the positions of the objects would have moved relative to an egocentric framework. Results demonstrated that LEC is critical for remembering the location of objects within an environment irrespective of the frame of reference in which they are presented.

On first inspection, this argues against the suggestion that LEC is preferentially involved in processing egocentric rather than allocentric spatial information and rather suggests that the principle role of LEC is the integration of features of an event. However, closer inspection of the data reveals that while LEC lesioned rats are impaired at the allocentric version of the OP task, relative to shams, they are still performing above chance. Interestingly, LEC lesioned animals’ ability to recognise a familiar OP association within an allocentric spatial framework took longer to develop than controls with significant memory for OP associations only occurring in the second half of the allocentric OP test trials. This shows that they can still use an allocentric frame of reference to remember the association of object with location even if it is not as efficient a process as it is in controls. In contrast, performance of the LEC lesioned rats on the egocentric version of the task is at chance. Clearly, while LEC is critical for the association of object and place, it is particularly important for tasks that involve processing of spatial information in an egocentric frame of reference.

How is it possible for rats with LEC lesions to remember the allocentric location of a previously experienced object? Previous experiments using complete LEC lesions had shown deficits in all associative recognition memory tasks (Chao et al., 2016; Kuruvilla and Ainge, 2017; Rodo et al., 2017; Van Cauter et al., 2013; Wilson et al., 2013a, 2013b). However, recent studies using more specific manipulations have demonstrated the existence of segregated functional circuits within the LEC. Leitner et al. (2016) showed that reelin and calbindin positive cells in layer 2 of LEC respond differently to odours and Vandrey et al. (2020) went on to show that specific inactivation of reelin positive cells in layer 2a of LEC results in impaired object–place–context memory while leaving object–context memory intact. While neither of these studies tested different spatial reference frames, they do demonstrate that LEC as a whole does not act as a functional unit but rather that it is made up of specialised sub-systems that can be functionally segregated. One potential explanation for the spared ability of LEC lesioned rats in this study to remember allocentric OP associations is that all of the lesioned rats had some residual LEC tissue within a consistent functional unit that could be used to support allocentric representations of the location of objects. Histological analysis of the lesions in this study showed that LEC lesioned rats did consistently have portions of the most ventromedial part of LEC still intact. However, while it is possible that ventromedial LEC has a specific role in allocentric spatial processing, this has not been investigated. Future studies could examine whether there are differences in processing egocentric versus allocentric frames of reference across this ventromedial–dorsolateral band of LEC.

We went onto examine whether lesions of LEC would produce a general deficit in spatial memory by examining performance on a non-associative reference memory task on the T-maze. The data show that rats with lesions of the LEC were not impaired and were actually better than controls in the early stages of the task. It is possible for rats to use either egocentric or allocentric frames of reference to solve the T-maze task and so it is interesting to examine whether the associative recognition tasks give an insight into how LEC lesions affected rats’ ability to remember the correct spatial location on the T-maze. Results from the OP experiments show that LEC animals do not remember the egocentric position of a previously seen object but do remember its allocentric position, albeit less well than shams. One interesting possibility here is that normal animals spend the first few days of T-maze training learning both an egocentric and allocentric frame of reference while LEC lesioned rats rely solely on the allocentric. This would mean they have less information to learn, making the task easier which could explain the increased T-maze accuracy in the LEC lesioned rats. Future studies could incorporate rotation trials where allocentric and egocentric frames of reference are placed into conflict to examine whether disruption of LEC changes strategy use.

Previous studies have suggested that LEC may process local spatial information rather than distal, global cues. We have previously shown that LEC lesioned rats can remember the position of food rewards in relation to global allocentric space but are impaired when learning the position of food relative to local cues (Kuruvilla and Ainge, 2017). The current data are consistent with this suggestion. In the allocentric OP task, animals are introduced to the environment from multiple different locations. This will mean that they encode the position of the objects within the allocentric framework of the testing arena but also the global spatial cues from the testing room. The fact that the LEC lesioned rats do better in the allocentric condition may be due to their ability to use the global distal room cues to solve the task.

The current data are consistent with unit recording data that have shown LEC responses to objects within the environment (Deshmukh et al., 2012; Deshmukh and Knierim, 2011; Tsao et al., 2013; Keene et al., 2016). These studies show that LEC neurons develop specific and consistent spatial tuning when objects are included in the local environment. Tsao et al. (2013) went on to show a small subset of LEC neurons encode the positions in which objects have been previously experienced. These ‘trace’ cells are a cellular correlate of OP memory and could support the behaviour reported in this study.

Previous studies have shown that LEC is one of four cortical hubs which receives extensive connections from the rest of cortex (Bota et al., 2015) putting it in an ideal position to integrate signals from multiple areas of the brain. This has two implications for the current study. First, it would be consistent with the primary role for LEC being in integration of features of an event and memory for the associations between these features. Second, close inspection of the anatomical inputs to LEC show a strong input from olfactory areas. Olfactory stimuli will be much more salient for local rather than global cues which would again be consistent with LEC having a role in the processing of local spatial features.

Overall, the current findings are in agreement with previous studies suggesting that the central role of LEC is in the integration of features that make up episodic memory (Beer et al., 2013; Chao et al., 2016; Hunsaker et al., 2013; Rodo et al., 2017; Van Cauter et al., 2013; Wilson et al., 2013a, 2013b). When the findings are combined with anatomical and electrophysiological studies, it creates a clear picture in which LEC encodes local, multimodal stimuli such as objects within the environment. This information can be used to support either egocentric or allocentric frames of reference.
